# A Novel Needle-Injectable Millimeter scale Wireless Electrochemical Glucose Sensing Platform for Artificial Pancreas Applications

**DOI:** 10.1038/s41598-019-53680-7

**Published:** 2019-11-22

**Authors:** M. Mujeeb-U-Rahman, Meisam. Honarvar Nazari, M. Sencan, William Van Antwerp

**Affiliations:** Integrated Medical Sensors Inc., 73 Overbrook, Irvine, CA 92620 USA

**Keywords:** Biomedical engineering, Design, synthesis and processing

## Abstract

Modern healthcare systems are under constant pressure to deliver personalized, effective care to billions of patients suffering from chronic non-communicable disease like diabetes. A closed-loop disease management system is an ideal solution for such patients. An example of this is an artificial pancreas for diabetes management. For safe and effective closed-loop disease management, the cost, size, longevity, warm-up time, and response speed need to match the performance of a healthy biological system (e.g. the pancreas). In this paper, a novel needle-injectable mm-size wireless sensing platform is presented to fulfill these requirements for an artificial pancreas by combining advanced microelectronics, nanotechnology and advanced biomaterial science. The proposed platform utilizes a sensor that is smaller than a sesame seed and provides fundamental advantages in terms of fast response speed, high accuracy, short warm-up time, and low cost of goods. Owing to these features, the system will enable true closed-loop glucose control (without any meal announcements and carbohydrate calculations), especially among infants and toddlers. The system has the potential to significantly improve diabetes management and in general chronic disease management for billions of patients.

## Introduction

Chronic diseases are now the leading cause of death worldwide, surpassing infectious diseases^1^. These changing disease patterns require a fundamentally different paradigm for a healthy society in the modern world. Instead of occasional treatment in a healthcare centre, people need more frequent, personalized, and automated treatments to manage their health. In the United States, about half of all adults (117 Million) have one or more chronic diseases^1^. A smart and scalable health system that can prevent complications and minimize hospitalization and adverse events is therefore essential. Diabetes alone is affecting more than 425 million people worldwide (30.3 million in US) with projected addition of 205 million by 2035^2^. In the US, complications related to diabetes lead to more than 14 million emergency room visits per year. Moreover, poor diabetes management results in an exorbitant burden of more than $322 Billion to the US economy annually. Long term complications due to diabetes are a leading cause of other severe health disorders such as stroke, cardiovascular issues, and kidney disease.

Closed-loop diabetes management i.e. the artificial pancreas is the technology of choice for diabetes management^3^. Accurate and fast glucose monitoring is essential for the success of the closed-loop artificial pancreas systems^4,5^. There are several ongoing research and development efforts in the field of artificial pancreas to enable a solution that can meet the clinical requirements. The three main aspects of these research efforts are improving the glucose sensor feedback, enhancing the efficacy and speed of insulin delivery, and developing a suitable algorithm to adjust the dose of insulin in an optimum manner^3,4^. Ultra-rapid, Smart, and even inhalable insulins are being developed currently to improve the actuation path of the closed-loop system^6^. The insulin delivery systems are also going through innovation in terms of smaller size, tubeless operation, and patch design making them easier and affordable^7^. The focus of this work is the optimization of the glucose sensor feedback, which like any control system, is a very important component required to obtain an efficient, safe, and reliable closed-loop solution. The major barriers in achieving true closed-loop glycemic control from the sensor point of view are improving hypoglycemia accuracy and minimizing sensor delay^8^. Long term CGM devices are an active area of research. Currently, devices from Senseonics and Glysens are most notable in this field^6^. However, these devices are rather large (bigger than a few centimeters) in size and require bulky electronics and expensive packaging, resulting in significant foreign body response making it difficult to achieve high accuracy in hypoglycemia and fast speed^9^. Hence, there is a need for a fundamentally new approach to sensor design which eliminates these limitations while still using clinically useful accuracy and speed.

In this paper, we present a glucose monitoring solution that is explicitly designed for artificial pancreas applications using modern Complementary Metal Oxide Semiconductor (CMOS) technology. The solution combines the benefits of wireless power harvesting and wireless communication using ultra-high-frequency radio (UHF) circuits and inductive-capacitive tuning circuits, the accuracy and versatility of integrated electrochemical sensing (clinically proven for glucose sensing), the precise control and low noise of on-chip sensor signal acquisition circuit, and the computational power of modern digital logic circuits, all on a single microchip that is much smaller than a sesame seed (0.6 mm × 3 mm × 0.1 mm). This local sensor control, signal processing and digitization enables digital communication of data from the sensor and makes it significantly faster and robust compared to other CGM sensors with remote processing of weak analog signals. The hair-thin (100 μm) structure of the system combined with a polymer coating makes it flexible to better integrate with the surrounding tissue and minimizes the foreign body reaction. Additionally, this small and flexible design reduces damage to the surrounding tissue during and after implantation and hence improves warm-up time, accuracy, stability, and response speed due to reduced foreign body response (FBR)^10,11^. Another fundamental advantage of this design is reduced system noise due to the elimination of wiring and enhanced signal-to-noise ratio (SNR) associated with miniaturized electrodes^12^, thus improving sensitivity and accuracy, especially in hypoglycemia. High SNR results in more accurate readings with less averaging and smaller electrodes enable faster electrochemical response speed^13^, both of which further enable the system to detect fast glucose dynamics. The system also minimizes blood-ISF lag by minimizing the damage to local microvascular environment as well as by fundamental improvements in device speed (less diffusion barrier owing to thinner foreign body capsule, less electrode capacitance) as a result of miniaturization and integration^14^. Together with the advancements in insulin delivery systems, this technology has the potential to enable widespread use of the artificial pancreas across a much broader patient population including infants and toddlers.

In the next sections, the details of the proposed system are presented followed by *in vivo* results from a 1 month study in a swine model.

## System Design

**Figure 1:**
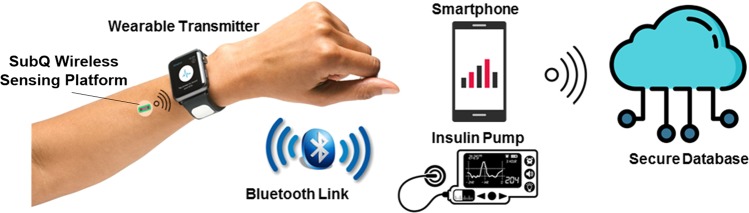
Components of the smart closed-loop diabetes management system 1. Subcutaneous wireless sensing platform 2. Wearable Transmitter 3. BLE Link from Transmitter to external reader 4. Smartphone and/or Insulin pump as external reader 4. Wireless Link to a secure Cloud 5. Secure Cloud.

**Figure 2:**
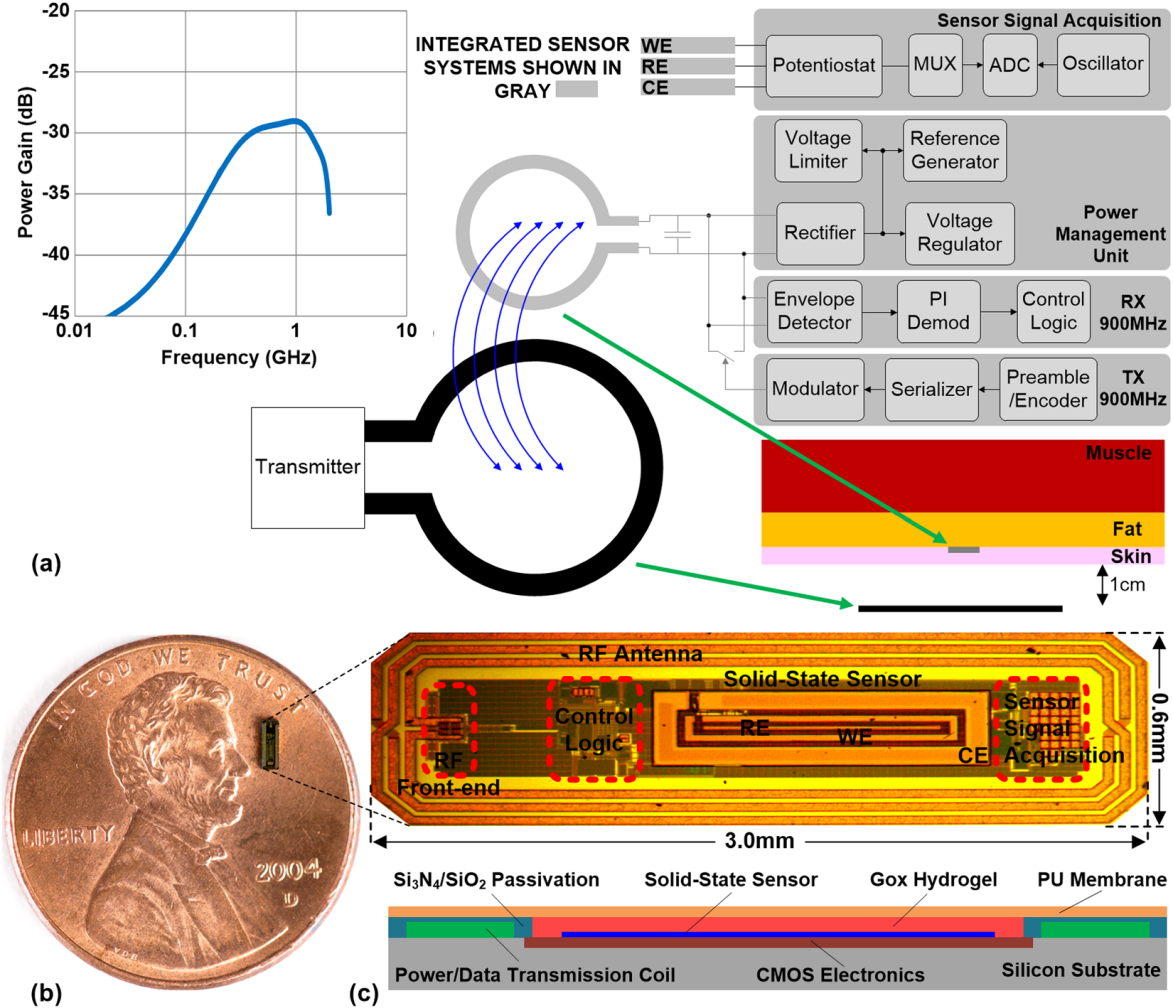
(**a**) Block diagram of the IMS Wireless Glucose Sensing System showing the external transmitter and the implantable sensor architecture. Coupled coils are used for wireless power and telemetry (**b**) The Integrated Wireless Sensing Platform on a U.S. one cent coin. (**c**) Monolithic sensor including CMOS electronics, solid-state sensor, enzyme hosting hydrogel, and biocompatible membrane.

The proposed wireless artificial pancreas system consists of the components shown in Fig. 1. The system uses the integrated wireless, electrochemical sensing platform to provide real-time glucose levels to a smart insulin infusion system (e.g. insulin pump or pen). The sensing platform is user-insertable through a custom injector and is wirelessly powered by and wirelessly communicates with a wearable, wireless transmitter (e.g. a smartwatch) using standard RFID technology at UHF frequency of 900 MHz ISM band (Fig. 2a). The transmitter utilizes standard Bluetooth low energy (BLE) interface to communicate with the insulin infusion systems and a smart reader (e.g. smartphone). The reader saves the data in a secure online database for further analysis and for personalization of treatment and management.

The focus of this work is the glucose monitoring platform and is discussed in further detail in the next sections.

### The sensing platform

The presented sensing platform utilizes electrochemical detection as the sensing mechanism due to its versatility, sensitivity and specificity, and fast response in complex physiological media like the interstitial fluid (ISF). A novel monolithically integrated microchip (Fig. 2b) is the practical realization of the platform. The top layer of the platform uses a solid-state electrochemical sensor with Pt working (WE) and counter electrodes (CE) and a Pt/PtOx reference electrode (RE), created by lithographic postprocessing^15^. In our work, we found Pt/PtOx to be a more suitable material due to its long term stability without the need of Ag in the body^15^. The electrodes are arranged as concentric rings in an overall area of 300 μm × 1,400 μm, as shown in Fig. 2c. With complete control and processing circuitry, and antenna coil for wireless operation, the overall subdermal platform is of the size of 600 μm × 3,000 μm × 100 μm. This is the smallest reported smart subdermal device with capabilities to perform real-time ISF monitoring at a footprint smaller than a sesame seed.

This integrated platform design minimizes size and enables monolithic integration, thus reducing foreign body response. The received RF power at the on-chip coil is converted into a DC voltage using a rectifier to provide power to the sensing circuitry. As part of the signal-acquisition circuit, an analog-to-digital converter (ADC) digitizes the sensor current with quantization sensitivity of 200pA once every millisecond (1 KSamples/sec), encodes and transmits the reading as a radio-frequency signal to the external transmitter via backscattering of the power signal^16,17^. This enables extremely low-power operation.

The sensor electronics consumes less than 5μW of power including the on-chip RF-to-DC power conversion. The power transfer efficiency from the external transmitter to the sensor through the inductive coupling link is about 0.1% (less than 30 dB power loss, as shown in Fig. 2a). This means 5 mW RF power transmission can fully energize the sensing platform. As there is no storage element on the sensing platform, its operation is solely dependent on the transmitted power from the external transmitter.

The planar semiconductor fabrication of the sensing platform allows us to obtain repeatable coatings by using thin film processing techniques such as spin coating. This together with repeatable semiconductor fabrication allows the system to be factory calibrated, relieving the patient from any reference measurements and making it easier to use the system.

The miniature design of the sensor is especially suited for use in infants and toddlers who can’t use current large wireless CGM sensors (e.g. Eversense by Senseonics). Additionally, the smart wireless transmitter eliminates the need for skin adhesives, making it easier for delicate skin of young and aging populations. These features are significant improvements for long term compliance of CGM and AP technologies across broad patient populations.

### Surface chemistry

Specific and sensitive detection of glucose is possible through a sensing reaction mediated by a glucose-specific enzymatic surface chemistry. The detection involves a two-step chemical reaction catalyzed by glucose oxidase (GOx) immobilized on the electrochemical sensor comprising of WE, CE, and RE:

1 $$\begin{array}{c}\beta -D-glucose+GOx-F{\rm{A}}D\to Gluono-\delta -lactone+GOx-F{\rm{A}}D{H}_{2}\\ GOx-F{\rm{A}}D{H}_{2}+{O}_{2}\to GOx-F{\rm{A}}D+{H}_{2}{O}_{2}\\ {H}_{2}{O}_{2}\mathop{\longrightarrow }\limits^{Pt\,Elctrode\,0.5V\,vs\,Ag/AgCl}{O}_{2}+2{H}^{+}+2{e}^{-}\end{array}$$

At the CE, reduction reaction(s) takes place to balance the WE current. The most common reaction is thought to be the reduction of dissolved oxygen.

2 $${O}_{2}+2{e}^{-}\to 2{O}^{-}$$

The enzyme is immobilized within a protein-based hydrogel that is in contact with the solid-state electrochemical sensor. In this work, GOx is immobilized on the working electrode within a BSA-based hydrogel using Glutaraldehyde as the crosslinking agent^15^. When glucose and oxygen diffuse into the hydrogel and encounter the enzyme, a chemical reaction takes place to generate hydrogen peroxide (H_2_O_2_) in the presence of the enzyme. This hydrogen peroxide is then detected at the platinum electrode biased at a positive potential versus the RE and generates a corresponding current that is proportional to the hydrogen peroxide concentration, as shown by Eqs 1 and 2.

To achieve linear response from the functionalized GOx based sensor, the stoichiometric ratio between oxygen and glucose needs to be regulated as oxygen is consumed during the enzymatic reaction and its concentration is low in the ISF compared to glucose. To mediate the imbalance between glucose and oxygen a thin membrane of Polyurethane (PU) is utilized which also provides sensor biocompatibility and mechanical integrity^18^.

### External transmitter and reader

The transmitter is used to wirelessly power the implant as well as to communicate with it using inductive near-field resonant magnetic coupling between two metal coils. It utilizes a coil of 2 cm diameter which is resonantly coupled to the on-chip antenna on the wireless implant^18^. The required wireless signals are generated by the transmitter using a UHF RFID transceiver. The 900 MHz ISM band enables sufficient power transfer from the transmitter to the sensor under the skin. The transmitter is battery powered (1WHr capacity) and is worn over the implant site. The transmitter uses Bluetooth low-energy (BLE) link to transmit data to the wireless reader (e.g. a smartphone) in a range of more than 10 meters. The BLE interface is also compatible with insulin pumps and pens that use standard CGM BLE profile. The transmitter battery can be charged in half an hour using microUSB interface. In continuous powering mode, this battery lasts for around 12 hours. The battery life can be extended by intermittent powering by adding a storage capacitor on-chip. Such capacitor can maintain energy to the sensor for several milliseconds. As the signal acquisition is performed in one millisecond, around 20% power duty cycling can be utilized to extend the battery life by 5 times.

### The applicator

The applicator is designed to insert the sensor in the subcutaneous space at a precise depth with minimal tissue damage. The current applicator utilizes a standard 18 gauge needle, although a 20.5 gauge needle can also be used as it fits a 0.6 mm wide sensor^19^. This is significantly smaller than the needle used for current wireless CGM product (Eversense by Senseonics) which has to fit a sensor with 3.5 mm diameter^20^. This also represents a significant improvement over previous IMS sensor that required a larger (12 gauge) needle for insertion^18^. This reduction is achieved by moving from a more uniform (square or circular) design to a rectangular design allowing for significant reduction in one dimension of the sensor. The new design allows significant advantages in terms of less implantation damage and less foreign body response, making it a more suitable candidate for long term *in vivo* studies presented in this paper. The sensor is loaded inside the applicator which is then placed on the skin and the needle is pushed at a shallow angle (less than 15°). Once the needle is inside the tissue (1 cm away from the incision site), the sensor is pushed out using a trocar through the needle. The applicator is then retracted. The sensor is attached to a thin, biocompatible, ultra-high molecular weight polyethylene (UHMWPE) thread that can enable sensor removal by pulling the thread out once the sensor life is over. Further details on implantation and extraction are presented in the methods section.

## Testing and Results

**Figure 3:**
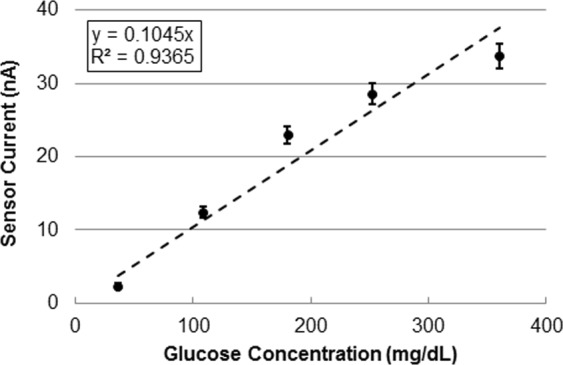
Sensor *in vitro* response.

The proposed system was first verified *in vitro* using spiked buffer testing. A typical *in vitro* curve is shown in Fig. 3 (n = 100 readings at each concentration). It shows a monotonic relationship between glucose concentration in the physiological range (36–360 mg/dl) and the sensor current. It also shows a low value of offset current and low noise levels.

**Figure 4:**
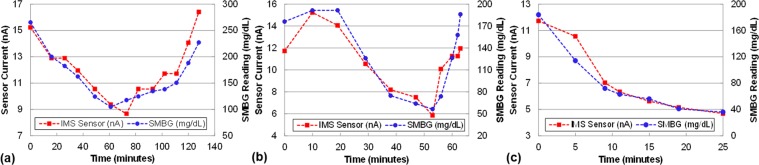
Presented sensor’s response in rat for one week (**a**) Day 1 (**b**) Day 3 (**c**) Day 5.

Next, an *in vivo* study was performed in two rats. The sensors were implanted using the custom IMS applicator and were read using the IMS wireless transmitter. The data was relayed to a wireless reader using BLE connection. Four devices were implanted in each of two rats (eight devices total) and tested for 5 days at the end of which the animals were sacrificed at the end of insulin-induced severe hypoglycemia within glucose and insulin tolerance test. No adverse medical events (infection, erosion, sensor migration, etc.) were encountered with any implant in any test. Results for first week for a typical sensor (Fig. 4) indicate the sensors were still functioning well after 5 days. The study also demonstrated fast sensor response with average blood-ISF lag of less than 2 minutes. This speed is significantly higher than that reported for other CGM sensors which reported 20 minutes in rats^21^. The study also revealed that the sensor could detect glucose accurately in deep hypoglycemia (<40 mg/dl) in real-time, indicating excellent accuracy at low glucose concentrations as desired.

### Swine study

Next, the functionality of the sensor for subcutaneous glucose measurement was tested in swine model due to appropriate skin structure and clinical and regulatory acceptability as a metabolic model. Four sensors and two sham devices (ultra-high molecular weight Polyethylene thread) were implanted in each of the one male and one female juvenile pig using the IMS applicator. One Dexcom G5 (Dexcom, San Diego, CA) and one Abbott Libre (Abbott Laboratories, Chicago, IL) continuous glucose monitoring device was inserted in the abdominal area in each swine per manufacturer’s instructions. Blood glucose values were obtained from an IV line using a Bayer Contour Next blood glucose monitor every five to ten minutes. Blood samples were also collected for plasma separation and subsequent YSI glucose measurement, every 15–20 minutes during the study. The sensors were inserted on the abdominal site as it is a good model for human skin. Sensor glucose readings were taken with the IMS wireless glucose monitoring system including the transmitter and the reader. Rapid acting insulin (Apidra, 1U/kg or as needed) and Dextrose 50 (1 mL/kg or as needed) were introduced via a second IV line to create glucose excursions. Insulin and glucose tolerance test studies were performed every other day for the first week. For animal health and integrity of IV lines, the studies were further apart afterwards. No adverse medical events (infection, erosion, sensor migration, etc.) were observed with any implant in any test. Also, no device movement was observed across all experiments. It was possible to locate sensors inside the living tissue with the wireless transmitter. Additionally, since the devices were implanted shallow, it was possible to locate them visually under bright white light. This together with no sensor movement ensures the system is safe to use. Tissue samples were collected for histology after performing euthanasia at the end of the study. Histology analysis was performed using H&E and Masson’s Trichrome stains.

**Figure 5:**
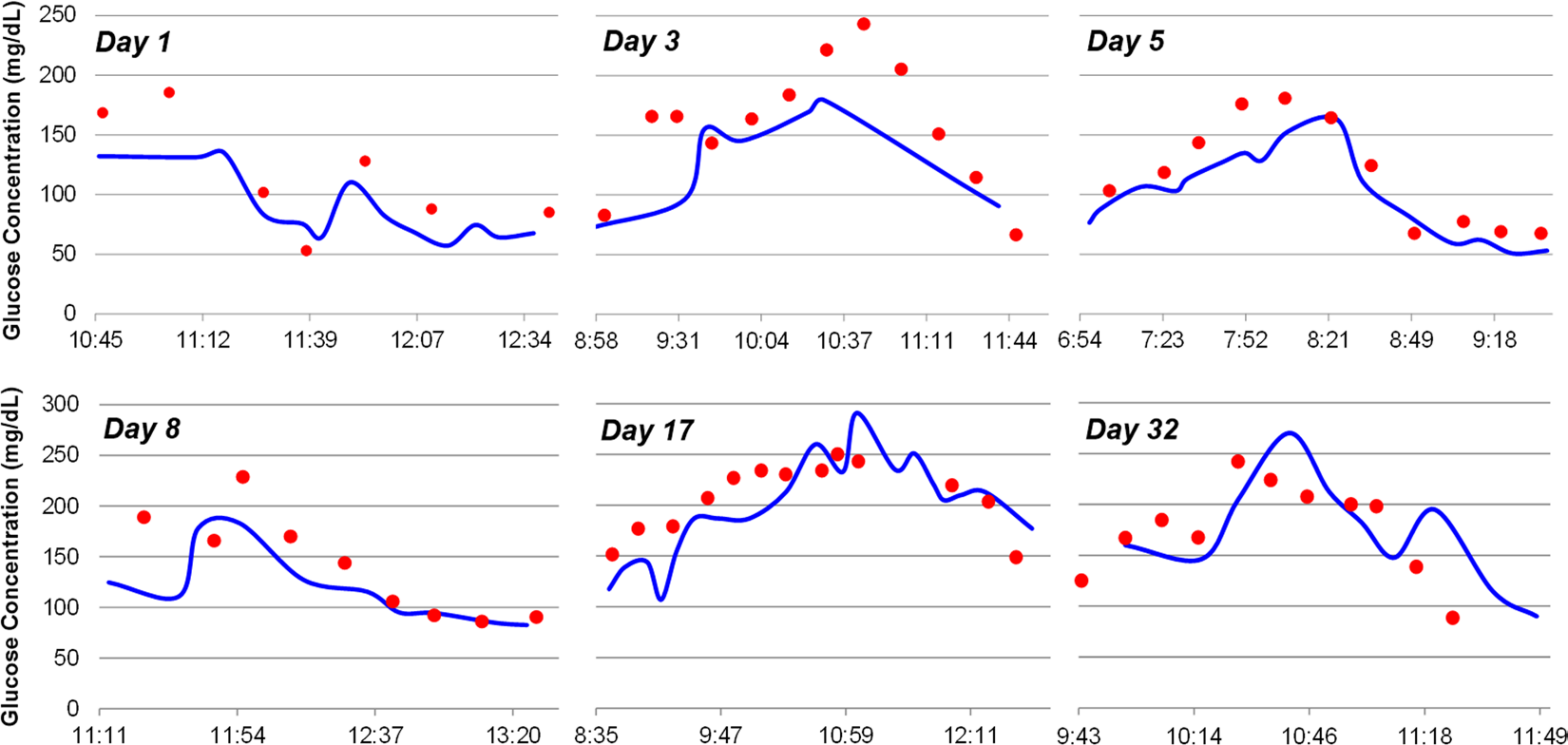
*In vivo* sensor operation in swine for more than a month. Blue line showing the sensor reading and red dots show the YSI readings.

Owing to the small size, minimally invasive insertion procedure, and wireless operation, it was expected that the sensor should experience sufficient perfusion and good tissue integration right after implantation. Hence, the warm-up time should be substantially short. To test this hypothesis, the IMS wireless sensor was tested 10 minutes after implantation. The sensor demonstrated stable and accurate reading, confirming the hypothesis (Fig. 5, day 1 reading). Single-point calibration was used for these readings on day 1 to adjust for changes in *in vivo* sensitivity compared to *in vitro* sensitivity. Reference glucose reading from blood glucose meter was used for this calibration. The sensors showed excellent response towards tracking glucose levels as low as 40 mg/dl (Fig. 5). The mean absolute relative difference (MARD) vs. YSI was calculated to be less than 10% for glucose values smaller than 180 mg/dl. This validates the excellent hypoglycemia accuracy as observed in rat studies earlier. The overall MARD for IMS sensor was calculated to be better than 12%. The G5 sensor and the Libre sensor demonstrated a MARD of 13%. The slightly worse than published performance in humans is hypothesized to be due the thicker fat tissue in the porcine model compared to humans. Other wireless CGM sensors in the market and under development were not used in testing (due to unavailability) but are expected to follow similar trends in accuracy i.e. the smaller sensor (Eversense by Senseonics) to be more accurate^20^ than the significantly larger (iCGM by Glysens) sensor^22^.

**Figure 6:**
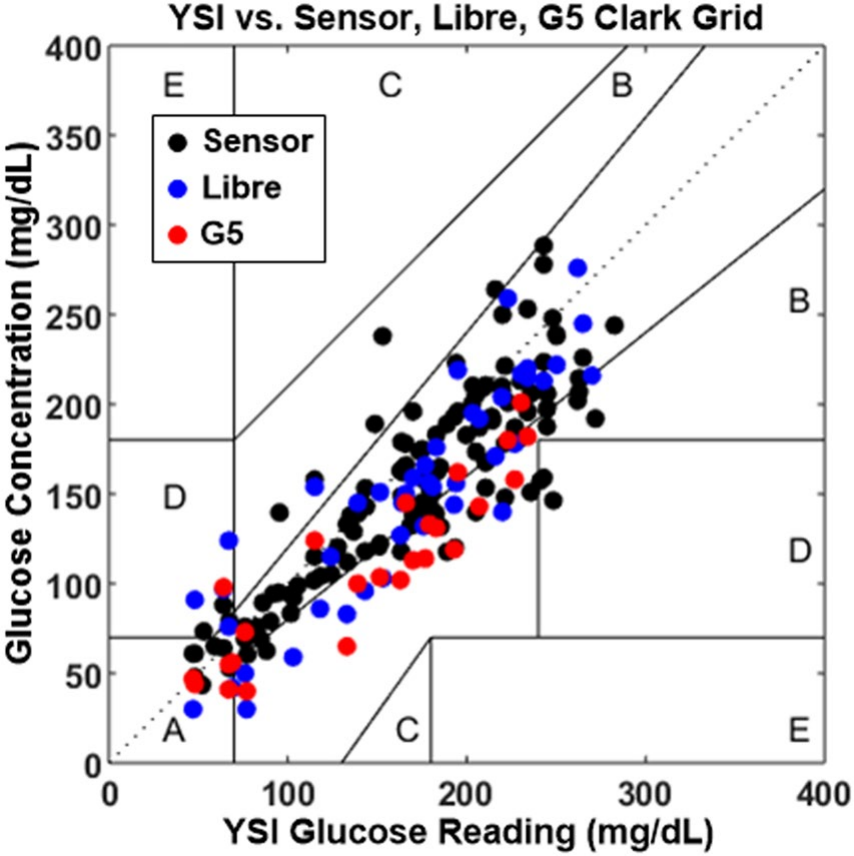
Clarke error grid indicates the proposed sensor accuracy *in vivo* for 1 month swine study.

**Figure 7:**
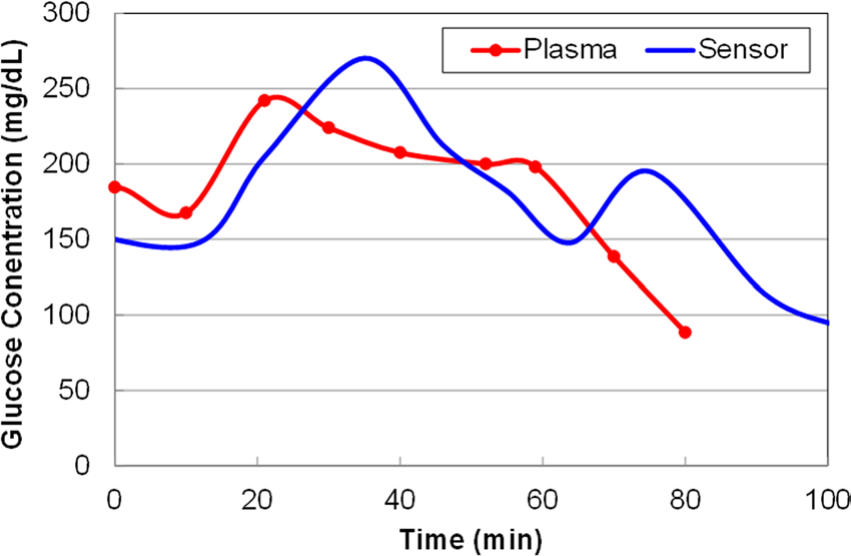
Sensor response during IVGTT excursion indicating the rising and falling of sensor response.

As the sensor was not wirelessly powered except during the testing, sensor response varied over time due to peroxide generation and accumulation, a known effect for glucose oxidase based sensors^7^. Therefore, 2 point calibration was required after day 8. For this process, two different blood glucose readings (separated by more than 20 mg/dl) were used to determine the slope and intercept of sensor response curve *in vivo*. Figure 5 summarizes the response of the presented sensor vs the YSI readings during the glucose excursion testing throughout the duration of the *in vivo* study. The results indicate that the sensor could reliably track glucose changes in the interstitial fluid and those matched well with the blood glucose readings (YSI). At some points, the sensor shows more dynamic variations than the YSI reading. However, this is expected as the YSI sampling rate (15–20 minutes for a reading) is lower than that of the wireless sensor. Clarke’s error grid analysis was performed on the data (Figs. 6 and 7). The result indicates that the presented wireless sensing platform had 96% points in regions A and B, 4% in region D, and no points in regions C and E (Table 1). The data points for G5 and Libre sensors are smaller in number as their wired transmitters were scratched off routinely by the animal despite attempts to protect the sensors with gauze and tape. Nonetheless, sufficient data points were collected from these commercial products to conclude that IMS sensor has the highest percentage of data points in region A, indicating excellent accuracy especially in hypoglycemia.

**Table 1:** Comparison of the proposed sensor with other CGM sensors.

Clarke Grid Region	A	B	C	D	E
IMS sensor	104 (78.2%)	24 (18%)	0	5 (3.8%)	0
G5	11 (44%)	13 (52%)	0	1 (4%)	0
Libre	27 (61.4%)	14 (31.8%)	0	3 (6.8%)	0

Response speed was measured for the presented wireless sensor by inducing a fast change in blood glucose level after achieving hypoglycemia using IV glucose injection. Faster SMBG sampling (5 minutes vs. 15 minutes for YSI) allowed us to capture the response speed of the sensor w.r.t blood glucose. An example of the sensor response delay to an intravenous glucose tolerance test (IVGTT) on day 32 during rising and falling of the glucose concentration is illustrated in Fig. 6. On average, the delay was observed to be 10.6 ± 8.9 minutes. The blood-sensor delay for the presented sensor in the first 8 days of the study was 5.25 ± 3.4 minutes (as shown in figure 7, close to the physiological limit of 5–6 minutes in humans^23^). This delay for the G5 sensor and the Libre sensor was measured to be greater than 12 minutes and 15 minutes respectively. In some cases, these commercial sensors took significantly long time (>20 minutes) to come out of “low” reading due to hypoglycemia after the SMBG/YSI reading had become greater than 50 mg/dl. Such readings were excluded from the calculation of MARD and delay for the commercial sensor but indicate the need for more accurate and faster sensor. The delay for wireless CGM sensors (e.g. Eversense by senseonics) is not well reported in literature but is expected to be even larger owing to their larger size. The IMS sensor on the other hand tracked hypoglycemia changes faster owing to its small size.

**Figure 8:**
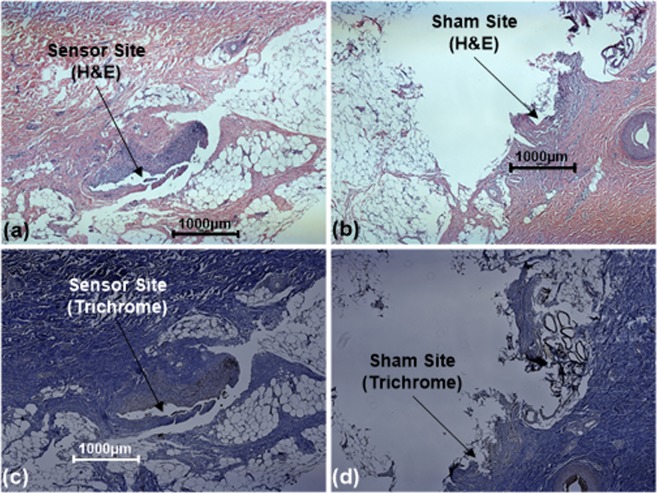
Histology of the tissue adjacent to the sensor implanted using the applicator (**a**,**c**), as well as the site only affected by applicator (**b**,**d**), using H&E and Trichrome staining.

Histology after 1 month (Fig. 8) demonstrated minimal to mild macrophage and collagen concentration around sensor site as compared to the sham site. The slight increase in the cellular and collagen concentration around the sensor can be attributed to mild foreign body response and tissue reaction to the accumulation of hydrogen peroxide generated by the sensor. This effect can be minimized by improving the biocompatible coating and by continuously consuming the excess peroxide using continuous powering.

## Discussion

The most relevant use of the proposed system is for patients in need of a fast glucose monitoring system suitable for closed-loop glycemic control as in an artificial pancreas. Unfortunately, the percutaneous wired sensors^24^ as well as the bulky wireless CGM systems possess significant blood-ISF delay in humans. This delay is caused by a combination of local disruption to the microvascular environment (longer diffusion time from farther capillaries), thicker foreign body encapsulation (longer diffusion time across the encapsulation), as well as the device designs (discrete designs and larger electrodes leading to bigger capacitance, and thicker membranes leading to slower diffusion). The presented sensor shows faster dynamics (5.25 minutes for first 8 days compared to >12 minutes for commercial products) which will enable faster feedback for the artificial pancreas leading to more accurate closed-loop control across all patient types. The sensor will thus eliminate the need for carbohydrate counting and meals announcement, which will enable true closed-loop artificial pancreas.

It is well established that smaller sensor electrodes lead to better signal to noise ratio^12^ and small implant size minimizes foreign body response. Both of these properties improve long-term accuracy. Moreover, the integration of electrochemical sensor, control, and processing circuitry minimizes transmission noise by enabling local digitization of weak analog signals. As a result, the presented small, semi-flexible, wireless sensing platform has higher accuracy over an extended time period (>1 month), and improved response in hypoglycemia as compared to current products. The sensor can measure glucose concentrations lower than 40 mg/dl *in vivo* and has MARD better than 10% (compared to 12% for commercial products) for most important glucose range (up to 180 mg/dl). Better hypoglycemia accuracy will be especially useful for insulin-dependent patients who need accurate glucose readings for proper dosing. This will also help minimize the risks associated with severe hypoglycemia as the sensor can accurately predict it before it actually happens.

Sensor lifetime is related to the extent of foreign body response and stability of the sensor chemistry. It is well established that the smaller the insertion trauma and the size of the implant, the smaller is the foreign body response. The dominant mechanism of chemistry degradation for glucose oxidase based sensors is the denaturing of glucose oxidase due to excess hydrogen peroxide generated during the transduction process. The foreign body response can be characterized by histology analysis and by post-explantation testing. As mentioned earlier, the histology analysis showed that the body response to the sensor was minimal. Also, the sensors didn’t show any improvement in sensitivity after being soaked in PBS solution at the end of the study. Therefore, hydrogen peroxide denaturing of glucose oxidase is likely to be the dominant reason behind the gradual decrease in the sensitivity. This degradation effect can be significantly minimized by continuously powering the sensor to avoid hydrogen peroxide accumulation. This continuous powering can be achieved by changing the form factor of current transmitter into a smaller device that can be easily worn or attached to the skin. With these modifications, the presented sensor is expected to last for a longer time (e.g. 6 months) as has been demonstrated for glucose oxidase based sensors^25^.

## Conclusion

In this paper, we presented an innovative integrated wireless glucose monitoring platform that will enable a wireless artificial pancreas. The sensing platform demonstrated following innovative features

1. Smallest form factor (0.196 mm^3^ i.e. smaller than a sesame seed) for a wireless electrochemical glucose sensing platform.
2. Best reported *in vivo* warm-up time (10 minutes).
3. Best reported *in vivo* hypoglycemia accuracy (accurate down to 40 mg/dl, <10% MARD) for wireless CGM.
4. Best reported blood-ISF lag (~5 minutes) in swine and even shorter (2 minutes) in rats for any CGM.
5. Longest reported lifetime (>1 month) for a user-insertable (due to small needle size) CGM.

In conclusion, the presented platform offers unprecedented accuracy, warm up time, response speed, and longevity for a continuous glucose monitoring system. The next steps are to develop a wearable transmitter that can be attached to the animal for continuous powering of the sensor. This is expected to decrease peroxide denaturing of the enzyme and hence increase sensor lifetime and decrease calibration requirements. Moreover, the sensor size will be further reduced to decrease the FBR even more and hence improve accuracy, response speed, and longevity. Further *in vivo* testing is required to gather more data on sensor performance, especially in comparison with other wireless sensing product available on the market (eversense) for a better comparison of accuracy and response speed. Nonetheless, the data gathered from *in vitro* studies, rat studies, and porcine study shows huge potential for such integrated needle-injectable wireless CGM products. Moreover, IMS is developing the next version of the sensor that would fit in an even smaller needle that is more common among diabetes patients, hence further reducing the adoption barrier and improving device performance.

## Materials and Methods

### CMOS postprocessing

The CMOS dies (5 mm × 5 mm) were lithographically post-processed to fabricate long-term stable electrochemical sensor electrodes by replacing CMOS top metal (Aluminum) with Platinum. Aluminum was chemically etched using Aluminum etchant type A (Transense Inc., Danver, MA). Next, photolithography using AZ5214e image reversal resist was performed to define the sensor pattern. This was followed by Titanium/Platinum thin film (10 nm/100 nm) sputtering and solvent liftoff. This was followed by mechanical grinding (for thinning 750 μm thick devices to 100–250 μm range) and mechanical dicing to separate individual sensors from the bigger die. The process was also repeated on 8 inch CMOS wafers to generate a large number of sensors.

### Sensor functionalization and packaging

Polyurethane was procured from Advansource Biomaterials (Wilmington, MA). All other chemicals were procured from MilliporeSigma (St. Louis, MO). Sensors were functionalized with a GOx hydrogel using BSA as an immobilization matrix and Glutaraldehyde as a crosslinking agent. A small volume (<10 nl) of BSA, GOx and 1% Glutaraldehyde mixture in PBS was deposited on sensor surface through spin coating. Sensors were left in ambient for 30 minutes for the gel to completely form followed by overnight soaking in PBS to remove any unbound gel. Next, 1% (by weight) polyurethane (PU) in THF with 1% (by weight) dexamethasone was coated on the sensor surface. Sensors were dried in ambient for 15 minutes after coating, followed by drying in a convection oven for 15 minutes. The sensors were left in ambient overnight and then sterilized using electron beam sterilization using 25 KGy dose.

### Implantation procedure

**Figure 9:**
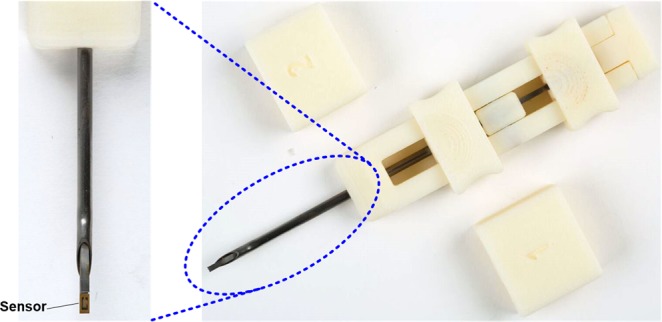
Sensor injection system (the applicator).

The animal studies were performed under a protocol approved by the University of California, Irvine Institutional Animal Care and Use Committee (IACUC approval # 16–003). All experiments were performed in accordance with relevant guidelines and regulations. Two sprague-dawley rats (both female) and two, one male and one female, juvenile Yucatan minipigs weighing approximately 25 kg at the beginning of study were used. The presented wireless sensors were implanted using the needle-based applicator (as shown in Fig. 9) sterilized with electron beam sterilization process. The applicator plastic parts were fabricated using a 3D printer and then assembled with an 18 gauge needle. The applicator needle length was about 2 inches to allow implantation of the sensor away from the wound site. Prior to implantation, animal skin was shaved followed by cleaning with sterile saline and betadine solution. The sterile sensor was loaded in the sterile applicator using sterile handling tools. The applicator was then used to make a small incision at a shallow angle (<15°) on the cleaned skin site. Through this incision, the applicator was extended under the skin to approximately 1 cm distance from the incision site, 1.5 mm deep from skin surface. The sensor was then mechanically dislodged from the applicator. The wound was covered with a small bandage followed by Tegaderm to prevent infections. In total, 4 sensors and 2 shams (length of UHMWPE thread, Teleflex Inc., Wayne, PA) were implanted in each of the two swine (only sensors were implanted in rats). All sensors were implanted in the abdominal area of the animals.

### Glucose tolerance tests

Glucose tolerance tests were conducted using intravenous injection of fast-acting insulin (Apidra, 1 unit/kg) and dextrose 50% (1 ml/kg) during the study sessions. Tail vein was used for rats to get blood samples for SMBG measurements. Intraperitoneal (IP) injections were used in the rats for glucose and insulin injections. In swine, I.V. line in one of the contralateral ears was used to inject glucose and insulin whereas the other contralateral ear was used to draw blood samples for glucose meter and YSI readings. The sensors were read using the wireless transmitter. The transmitter was taped to the skin for continuous readout during the study session. Blood samples for YSI readings for the swine study were taken every fifteen 15 minutes from ear veins. These blood samples were allowed to clot for 30 minutes in ice and spun at 10,000 rpm for 10 minutes to separate plasma. The plasma samples were pipetted out and read directly with a YSI 2300 (YSI Inc., Yellow Springs, OH) instrument. Three readings were taken for each YSI sample and their mean was used for further analysis.

### Sensor calibration

For statistical characterization of sensor accuracy, a typical sensor was first tested in a series of known glucose concentrations {*g*_1_*, g*_2_*, …, g*_*m*_} to determine the corresponding sensor currents {*i*_1_*, i*_2_*, …, i*_*m*_}. Curve fitting was used to determine the concentration-current relationship. Either a first-order linear equation or a second order equation was used based upon sensor linearity. Glucose concentrations in the known solution were measured using YSI 2700 benchtop glucose analyzer. Once calibration curve is established, sensor currents are measured in random glucose solutions (*in-vitro*), or in live animals (*in vivo*), and converted to glucose values CGM using the said curve. The resulting glucose values were tested against the actual glucose concentration as determined with the YSI. The difference in the reading was used to calculate the mean absolute relative difference (MARD). Factory calibration was used for the swine study for first 8 days (single point calibration was used to determine nA to mg/dL conversion constant). 2-point calibration (assuming linear current-concentration relationship) was used afterwards. 2 different points from SMBG readings were used to determine the slope and intercept of the sensor response curve and hence the linear equation. The same equation was used to predict glucose concentration from sensor current for all other sensor readings (Glucose = slope* current + intercept).

### Delay measurements

For blood-ISF delay measurements, SMBG readings were used for reference as faster sampling was possible compared to the YSI. Delay was defined as the time difference between peak reading with SMBG and the corresponding peak reading in the sensor response during glucose excursion. Delay across sensor lifetime was processed to calculate mean and standard deviation.

### Histology

Tissue samples were collected following euthanasia and stored in formalin. Sensors were carefully removed from these samples before putting these in formalin. After 3–7 days, standard histology techniques were used to fix the samples, cut multiple sections, and stain those with H&E and Masson’s Trichrome. The stained slides were imaged using a Nikon top-illumination microscope. The resulting images were analyzed to compare the concentration of cells and collagen around the implant site as compared to sham sites. The stained samples were also inspected to measure the implantation depth of different sensors used in the study.

## References

[1] Organization, W. H. & Others. World health statistics 2018: monitoring health for the SDGs, sustainable development goals. (2018).

[2] Cho NH (2018). IDF Diabetes Atlas: Global estimates of diabetes prevalence for 2017 and projections for 2045. Diabetes Res. Clin. Pract..

[3] Farrington C (2015). The artificial pancreas: challenges and opportunities. Lancet Diabetes Endocrinol.

[4] Christiansen SC (2017). A Review of the Current Challenges Associated with the Development of an Artificial Pancreas by a Double Subcutaneous Approach. Diabetes Ther..

[5] Jr, P. *et al*. Improving the clinical value and utility of CGM systems: issues and recommendations: a joint statement of the European Association for the Study of Diabetes and the American Diabetes Association Diabetes Technology Working Group. *Yearbook of Paediatric Endocrinology*, 10.1530/ey.15.10.9 (2018).

[6] Bailey TS, Walsh J, Stone JY (2018). Emerging Technologies for Diabetes Care. Diabetes Technol. Ther..

[7] Kleppe K (1966). The effect of hydrogen peroxide on glucose oxidase from Aspergillus niger. Biochemistry.

[8] Huyett, L. M., Dassau, E., Zisser, H. C. & Doyle, F. J. The impact of glucose sensing dynamics on the closed-loop artificial pancreas. In *2015 American Control Conference (ACC)*, 5116–5121 (2015).

[9] Allen, N. & Gupta, A. Current Diabetes Technology: Striving for the Artificial Pancreas. *Diagnostics (Basel)* **9** (2019). 10.3390/diagnostics9010031 PMC6468523 30875898

[10] Veiseh O (2015). Size- and shape-dependent foreign body immune response to materials implanted in rodents and non-human primates. Nature Materials.

[11] Ward WK, Slobodzian EP, Tiekotter KL, Wood MD (2002). The effect of microgeometry, implant thickness and polyurethane chemistry on the foreign body response to subcutaneous implants. Biomaterials.

[12] Morgan DM, Weber SG (1984). Noise and signal-to-noise ratio in electrochemical detectors. Anal. Chem..

[13] Forster RJ (1994). Microelectrodes: new dimensions in electrochemistry. Chem. Soc. Rev..

[14] Helton KL, Ratner BD, Wisniewski NA (2011). Biomechanics of the Sensor-Tissue Interface—Effects of Motion, Pressure, and Design on Sensor Performance and Foreign Body Response—Part II: Examples and Application. J. Diabetes Sci. Technol..

[15] Mujeeb-U-Rahman, M., Nazari, M. H., Sencan, M. & Scherer, A. Fabrication of implantable fully integrated electrochemical sensors. *US Patent* (2015).

[16] Nikitin PV, Rao KVS (2006). Theory and measurement of backscattering from RFID tags. IEEE Antennas Propag. Mag..

[17] Nazari, M. H., Mujeeb-U-Rahman, M. & Scherer, A. An implantable continuous glucose monitoring microsystem in 0.18 µm CMOS. In *2014 Symposium on VLSI Circuits Digest of Technical Papers* 1–2 (2014).

[18] Mujeeb-U-Rahman M, Nazari MH, Sencan M (2019). A novel semiconductor based wireless electrochemical sensing platform for chronic disease management. Biosensors and Bioelectronics.

[19] Syringe Needle Gauge Chart. *Sigma-Aldrich* Available at, https://www.sigmaaldrich.com/chemistry/stockroom-reagents/learning-center/technical-library/needle-gauge-chart.html, (Accessed: 19th May 2019).

[20] Kropff J (2017). Accuracy and Longevity of an Implantable Continuous Glucose Sensor in the PRECISE Study: A 180-Day, Prospective, Multicenter, Pivotal Trial. Diabetes Care.

[21] Croce RA (2013). A miniaturized transcutaneous system for continuous glucose monitoring. Biomed. Microdevices.

[22] Lucisano JY, Routh TL, Lin JT, Gough DA (2017). Glucose Monitoring in Individuals With Diabetes Using a Long-Term Implanted Sensor/Telemetry System and Model. IEEE Trans. Biomed. Eng..

[23] Basu A (2013). Time lag of glucose from intravascular to interstitial compartment in humans. Diabetes.

[24] Sinha M (2017). A Comparison of Time Delay in Three Continuous Glucose Monitors for Adolescents and Adults. J. Diabetes Sci. Technol..

[25] Gough DA, Kumosa LS, Routh TL, Lin JT, Lucisano JY (2010). Function of an implanted tissue glucose sensor for more than 1 year in animals. Sci. Transl. Med..

